# In-Plane Strain Tuned Electronic and Optical Properties in Germanene-MoSSe Heterostructures

**DOI:** 10.3390/nano12193498

**Published:** 2022-10-06

**Authors:** Qing Pang, Hong Xin, Ruipeng Chai, Dangli Gao, Jin Zhao, You Xie, Yuling Song

**Affiliations:** 1 College of Science, Xi’an University of Architecture and Technology, Xi’an 710055, China; 2 Shaanxi Key Laboratory of Nano Materials and Technology, Xi’an University of Architecture and Technology, Xi’an 710055, China; 3 College of Science, Xi’an University of Science and Technology, Xi’an 710054, China; 4 College of Physics and Electronic Engineering, Nanyang Normal University, Nanyang 473061, China

**Keywords:** germanene, MoSSe monolayer, heterostructures, first-principles calculation

## Abstract

DFT calculations are performed to investigate the electronic and optical absorption properties of two-dimensional heterostructures constructed by Janus MoSSe and germanene. It is found that a tiny gap can be opened up at the Dirac point in both Ge/SMoSe and Ge/SeMoS heterostructures, with intrinsic high-speed carrier mobility of the germanene layer being well preserved. An n-type Schottky contact is formed in Ge/SMoSe, while a p-type one is formed in Ge/SeMoS. Compared to corresponding individual layers, germanene-MoSSe heterostructures can exhibit extended optical absorption ability, ranging from ultraviolet to infrared light regions. The position of the Dirac cone, the Dirac gap value as well as the position of the optical absorption peak for both Ge/SMoSe and Ge/SeMoS heterostructures can be tuned by in-plane biaxial strains. It is also predicted that a Schottky–Ohmic transition can occur when suitable in-plane strain is imposed (especially tensile strain) on heterostructures. These results can provide a helpful guide for designing future nanoscale optoelectronic devices based on germanene-MoSSe vdW heterostructures.

## 1. Introduction

The discovery of graphene and its successful applications in the current nano-industry have inspired great interest in exploring new two-dimensional (2D) materials with novel properties [1,2]. Recently, one kind of 2D material, named 2D Janus transition metal dichalcogenides (JTMD), has gradually become a research hotspot [3,4,5,6]. Due to their asymmetric structure, 2D JTMD materials have an intrinsic dipole perpendicular to the surface, which leads to many new properties, including a strong Rashba effect [7], out-of-plane piezoelectric polarization [8,9]^,^ and better performance in photocatalysts for water splitting [10,11]. As a prototype, the Janus MoSSe monolayer has been fabricated either from selenization of MoS_2_ or sulfurization of MoSe_2_ [12,13]. It is a direct semiconductor with a band gap of ~1.5 eV, and its band gap, surface dipole and carrier mobility can be tuned by different stacking patterns and thicknesses [14]. Single transition metal (TM) atom adsorption can introduce magnetism into Janus MoSSe, rendering them good magnetic photocatalysts, not only suitable for the recycling of efficient photocatalysts but also with potential for spintronic applications [15]. By means of strain modulation, defect engineering and TM functionalization, the Janus MoSSe monolayer can also exhibit ultrahigh sensitivity in gas detection, which suggests that it is an ideal material for constructing superior gas sensors [16,17,18]. Moreover, theoretical studies have predicted that Janus MoSSe can act as a potential anode material for lithium-ion batteries [19,20].

Stacking different 2D materials to form a vertical van der Waals (vdW) heterostructure has proven to be an effective way of designing new optoelectronic devices [21,22,23]. Due to weak interlayer coupling, the vdW heterostructures could not only retain the intrinsic advantages of their individual components, but also exhibit new properties. This raises interest in designing novel heterostructures based on Janus monolayer materials [24]. By considering different stacking modes and sulfur atomic orders, Idrees et al. predicated that MoSSe-WSSe, MoSe-WSeTe and MoSTe-WSTe heterojunctions are all type II semiconductors with edges of conduction band and valence band located outside the redox region, making them suitable candidates for water splitting [25]. A vdW heterojunction composed of blue phosphorus and a Janus MoSSe monolayer shows different types of band alignment (type I or type II) depending on its stacking models, and the band gap and band alignment can be effectively modulated by an applied electric field and strain [26]. In MoSSe-AlN and MoSSe-GaN heterojunctions, Yin et al. found that intrinsic dipole, acting as an auxiliary booster for photo-excited carriers, can induce suitable band alignment for water splitting in the visible-infrared (IR) region [27]. Theoretical studies indicate that the high-speed carrier mobility of graphene and the prominent ultraviolet (UV) to visible light absorption of JTMD can still be maintained after the formation of vertical heterojunctions, and the electronic and optical properties of graphene-JTMD heterojunctions can be adjusted by interlayer distance, external electrical field, as well as in-plane strain, thus enriching their applications in tunable nanoelectronic devices [28]. In graphene-MoSSe heterojunctions, Yu et al. found that a transition from n-type Schottky contact to Ohmic contact can be realized by an applied external electric field and in-plane tensile strain [29], and recent research by Wang et al. reported that single-vacancy defects (S or Se) can not only reduce the Schottky barrier height (SBH) of the heterojunction but also improve its carrier mobility [30]. As promising anode materials for Li-ion batteries, density-functional theory (DFT) calculations indicate that graphene-MoSSe heterostructures possess good structure stability, a low diffusion barrier, superior Li-ion conductivity and high mechanical stiffness [31,32].

As a germanium analog to graphene, germanene shows similar electronic properties to graphene and has been experimentally fabricated on both metallic and ceramic substrates [33,34]. Its high-speed intrinsic carrier mobility [35], larger spin-orbit coupling gap [36], good compatibility with Si-based technology [37], and good sensing characteristics for certain gases [38] endorse it as a promising material for constructing nanoelectronic devices. However, the absence of a gap limits its application in nanoelectronics, such as field effect transistors (FETs). Previous studies have demonstrated that hetero-physisorption [39,40] or formation of vertical vdW heterojunctions [41,42] are feasible ways of solving this problem; these mechanisms can not only open up a gap at the Dirac point but also keep the shape of the Dirac cone of germanene. The electronic properties of germanene are also strongly influenced by the substrate. For instance, when germanene is on a graphene or graphite substrate, it is reported that weak interaction at the interface breaks the sublattice symmetry of the germanene overlayer, thus opening a gap at the Dirac point in germanene, and rotation between the overlayer and substrate also results in a mapping of the Dirac point from K/K’ to Γ in the Brillouin zone (BZ) [43,44]. 

Motivated by the intriguing achievements in 2D JTMD and germanene described above, it is worthwhile exploring the properties and applications of vertical heterojunctions based on semimetal germanene and JTMD monolayers. Thus, in the present work, we establish two types of vertical heterostructures stacked by germanene and Janus MoSSe monolayer, and systematically investigate their structures, electronic and optical properties based on DFT calculations with vdW correction. We found that two components in both types of heterostructures can complement each other well, and a tiny gap can be opened up at the Dirac point with the high carrier mobility of germanene being well-preserved. The heterostructures also exhibit extended light absorption (including UV, visible and IR regions) compared to the corresponding individual layers. Moreover, the position of the Dirac cone, the Dirac gap value, the SBH, as well as the optical absorption peak can be well tuned through applied in-plane biaxial strains. Our results suggest germanene-MoSSe heterostructures should be promising materials for designing future nanoscale optoelectronic devices. 

## 2. Theoretical Methods and Models 

All total energy and electronic structure calculations are implemented in the Vienna ab initio simulation package (VASP) based on projector-augmented wave (PAW) pseudopotentials [45,46]. The generalized gradient approximation with the exchange-correction function of Perdew–Burke–Ernzerhof form (GGA-PBE) is utilized for structure optimization [47]. To improve the description of vdW interactions, the semi-empirical DFT-D2 method by Grimme is also used [48]. The kinetic energy cutoff is set to be 400 eV for plane wave basis expansion, and convergence criteria of electronic self-consistent energy and atomic force are, respectively, set to 10^−5^ eV and 0.01 eV. A slab model with periodic conditions is adopted for the simulation. To eliminate the interaction between the neighbor slabs, a vacuum layer of 20 Å is added in the direction perpendicular to the surface (*Z* direction). The first Brillouin zone (BZ) is sampled by Γ-centered (7 × 7 × 1) and (11 × 11 × 1) Monkhorst-Pack [49] grids for structure relaxation and electronic properties calculations respectively. In addition, the frequency-dependent dielectric matrix [50] is also calculated to characterize the optical properties of the heterostructures, and the imaginary part is determined by a summation over empty states using the following equation:

(1) $$\varepsilon_{2}^{\left(\alpha \beta \right)}=\frac{4\pi^{2}e^{2}}{\Omega }\underset{q\to 0}{\lim}\frac{1}{q^{2}}\sum_{c,v,k}2w_{k}\delta (\varepsilon_{ck}-\varepsilon_{vk}-\omega )\times \langle u_{ck+e_{\alpha }q}|u_{vk}\rangle \langle u_{ck+e_{\beta }q}|{u_{vk}\rangle }^{*}$$

where Ω is the volume of the cell, the indices c and v refer to conduction and valence band states, respectively, $w_{k}$ represents the k point weight, $\varepsilon_{ck}$ and $u_{ck}$ are the eigenvalues and the cell periodic part of the wavefunctions at the k point, and ω is the angular frequency of light. In our calculations, a good number of empty conduction bands (about 2 times more than the number of valence bands) are adopted and the number of frequency grid points is increased to 2000. 

The hexagon lattice parameters of monolayer MoSSe and free-standing germanene after optimizations are $a_{\mathrm{MoSSe}}$ = 3.254 Å and $a_{Ge}$ = 4.059 Å, respectively. The layer height of MoSSe and the buckling height of germanene are 3.225 and 0.689 Å, respectively. These values are all consistent with previous reports [12,33,34]. To construct the vertical germanene-MoSSe heterostructures, we use a (4 × 4) supercell of germanene adhered to a (5 × 5) supercell of MoSSe monolayer (containing 32 Ge, 25 Mo 25 S and 25 Se atoms), resulting in a negligible lattice mismatch of 0.21% between the germanene and MoSSe layer. During calculations, the initial lattice constant of the germanene-MoSSe heterostructure is set to 16.253 Å (the averages of 4$a_{\mathrm{Ge}}$ and 5$a_{\mathrm{MoSSe}}$), and both the lattice constants and atomic geometry are fully relaxed. To evaluate the stability of the heterostructures, the binding energy ($E_{b}$) between the germanene and MoSSe monolayer is calculated as:

(2) $$E_{b}=\left[E_{\mathrm{Ge}-\mathrm{MoSSe}}-(E_{\mathrm{Ge}}-E_{\mathrm{MoSSe}})\right]/A$$

where $E_{\mathrm{Ge}-\mathrm{MoSSe}}$, $E_{\mathrm{Ge}}$ and $E_{\mathrm{MoSSe}}$ are the total energies of the heterostructure, the freestanding (4 × 4) germanene and the pristine (5 × 5) MoSSe monolayer, respectively, while A is the interface area of the heterostructure. 

## 3. Results and Discussions

**Figure 1:**
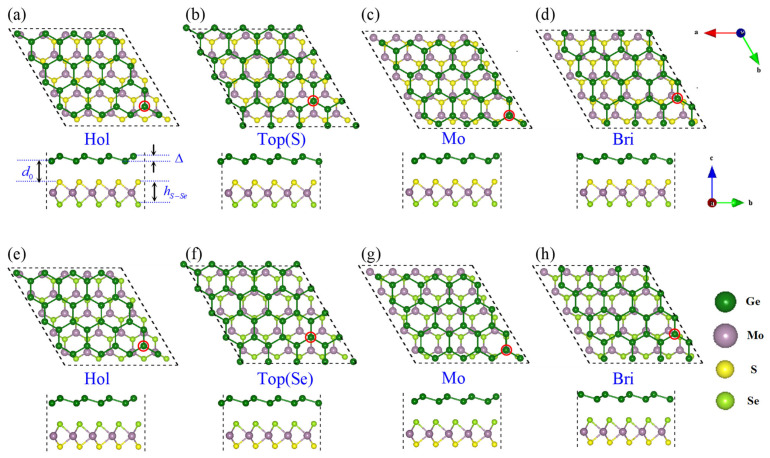
Top and side views of representative configurations of Ge/SMoSe (**a**–**d**) and Ge/SeMoS (**e**–**h**) heterostructures. The interlayer $d_{0}$, buckling height of germanene Δ, and height of the MoSSe layer $h_{S-Se}$ are labeled as an example in (**a**).

Because of the different atoms (S or Se) on each side of the MoSSe monolayer, two typical types of the germanene-MoSSe heterostructures are established, named Ge/SMoSe and Ge/SeMoS. For each type, four representative stacking patterns are considered, i.e., (1) one Ge atom in the germanene supercell at the center above a hexagon of MoSSe supercell, with the positions of other Ge atoms then to be determined (Hol); (2) one Ge atom directly above a S atom or Se atom in the MoSSe layer (Top); (3) one Ge atom directly above a Mo atom in the MoSSe layer (Mo); and (4) one Ge atom above a S(Se)-Mo bond in the MoSSe layer (Bri) (shown clearly in Figure 1). The calculated binding energies $E_{b}$ and structure parameters (including lattice constant $a_{0}$, interlayer distance $d_{0}$, buckling height of germanene Δ, and height of MoSSe layer $h_{S-Se}$) for these configurations of germanene-MoSSe heterostructures are listed in Table 1. It can be seen that the binding energies ($d_{0}$) for both Ge/SMoSe and Ge/SeMoS are not sensitive to the stacking patterns of the atomic layers, and the calculated structural parameters for different configurations are also very similar. The binding energies of Ge/SMoSe and Ge/SeMoS are mainly located at the region −22~−20 meV, which is comparable to other vdW heterostructures [51]. The Hol configuration is the most energetically favorable structure for the Ge/SMoSe type, while the Mo configuration is the most energetically favorable for the Ge/SeMoS type. Therefore, further calculations and discussions are based on these two stable configurations for simplicity. The interlayer distance $d_{0}$ (labeled in Figure 1a) for the favored Ge/SMoSe and Ge/SeMoS are 2.924 and 3.068 Å, respectively, much shorter than those of graphene-MoSSe heterostructures (~3.36 Å) [29,32], indicating relatively stronger interaction between the germanene and MoSSe layer. Due to the interlayer interaction, the buckling height of germanene Δ, and the height of the MoSSe layer $h_{S-Se}$ are also slightly increased to ~0.72 Å and ~3.24 Å. 

**Figure 2:**
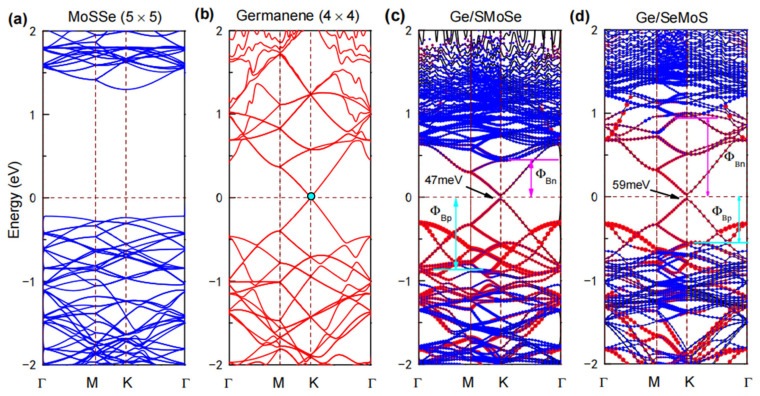
The band structures of (**a**–**d**) heterostructures. In Figure 2c,d, the contributions of MoSSe and germanene in the heterostructures are marked by solid blue and red circles, respectively.

**Figure 3:**
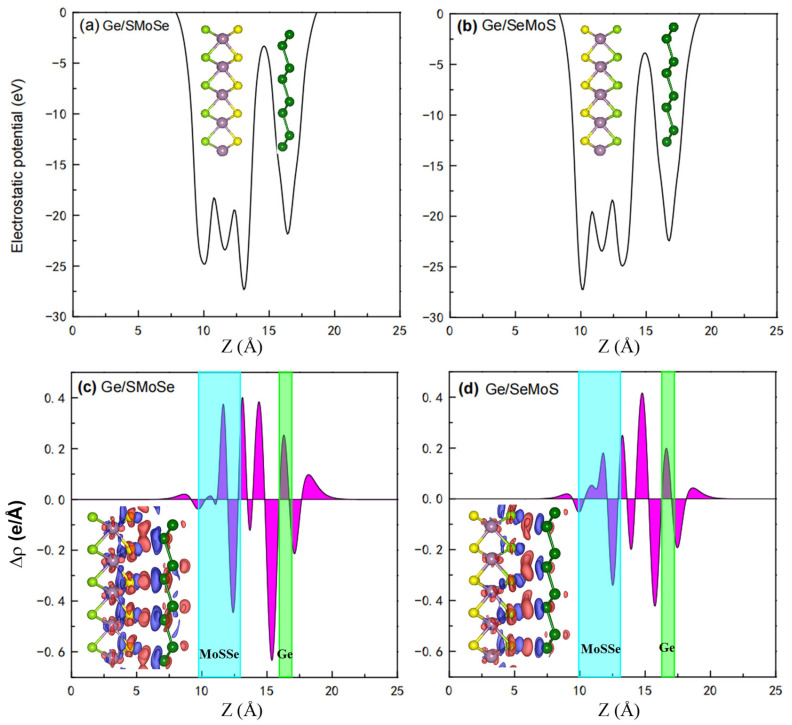
Electrostatic potential of (**a**,**b**) Ge/SeMoS. The corresponding planar-averaged differential charge density Δρ are shown in (**c**,**d**) with locations of component layers marked out. The insets in (**c**,**d**) are the 3D isosurfaces of charge density difference, where red and blue areas represent electron accumulation and depletion, respectively.

We now consider the electronic properties of germanene-MoSSe heterostructures. Figure 2 depicts the band structures of a free-standing (4 × 4) germanene supercell, a pristine (5 × 5) MoSSe supercell and the favored Ge/SMoSe and Ge/SeMoS heterostructures. As predicted in previous research [12,32,33], the MoSSe monolayer is a *p*-type semiconductor with a band gap of ~1.54 eV (Figure 2a), while germanene is a semimetal with electron and hole bands forming a nearly linear crossing at the Fermi level (Figure 2b). From Figure 2c,d, we can clearly see that the electronic characteristics of germanene-MoSSe heterostructures seem to be a combination of those of the MoSSe monolayer and germanene. The Dirac cone of the germanene layer can be well-preserved in the heterostructures, which indicates that the heterostructures also have high-speed carrier mobility. As in the case of germanene on a graphene or graphite substrate [43,44], due to the breaking of the sublattice symmetry of the germanene layer driven by weak vdW interaction at the germanene-MoSSe interface, the Dirac point (marked in Figure 2b) at *K* point also opens up to form a tiny energy gap in the heterostructures (47 meV for Ge/SMoSe and 59 meV for Ge/SeMoS), thus being convenient for current field effect transistor (FET) applications. However, by comparison to the pristine MoSSe layer, the band gap of the semiconductor layer for the heterostructure (measured from the weighted band structures of MoSSe contribution in Figure 3c,d) becomes narrower, especially for Ge/SMoSe (~1.32 eV), suggesting non-negligible hybridization between the component layers. In addition, we notice that the Fermi level is close to the conduction band minimum (CBM) of the semiconducting MoSSe layer in Ge/SMoSe, while close to the valence band maximum (VBM) in Ge/SeMoS. According to the Schottky–Mott model for the metal/semiconductor interface [52], an n-type Schottky contact at Ge/SMoSe is formed with an n-type barrier ($\Phi_{\mathrm{Bn}}$) of ~0.45 eV, while a p-type one at Ge/SeMoS with a p-type barrier ($\Phi_{\mathrm{Bp}}$) of ~0.54 eV ($\Phi_{\mathrm{Bn}}$ and $\Phi_{\mathrm{Bp}}$ also marked in Figure 2c,d). The electronic band structures are also calculated for other configurations of each type (see Figure S1 from Supplementary Materials), which are not sensitive to the stacking patterns. 

To better understand the charge transfer mechanism and bonding character between the MoSSe monolayer and germanene, the electrostatic potential and planar-averaged differential charge density along the *Z* axis of Ge/SMoSe and Ge/SeMoS are calculated, and the results are shown in Figure 3. It can be clearly seen in Figure 3a,b that the MoSSe layer possesses a much deeper electrostatic potential than germanene, leading to an electrostatic potential drop across Ge/SMoSe and Ge/SeMoS. Such a potential drop can induce an electrostatic field across the interface of the heterostructures, which may affect their charge redistribution. The differential charge density isosurface plots are also illustrated as insets in Figure 3c,d. We can see obvious charge depletion (blue) on the Ge atoms close to the MoSSe layer and charge accumulation (red) in the region between the component layers, especially for Ge/SMoSe. Therefore, a somewhat covalent bonding is formed in the heterostructures. The electron transfer (Δq) from germanene to the MoSSe layer is also evaluated by Bader charge analysis [53]. The results show that there is relatively greater electron transfer in Ge/SMoSe (1.241 e) than in Ge/SeMoS (0.809 e), and thus stronger interlayer interaction and hybridization in Ge/SMoSe than in Ge/SeMoS. 

**Figure 4:**
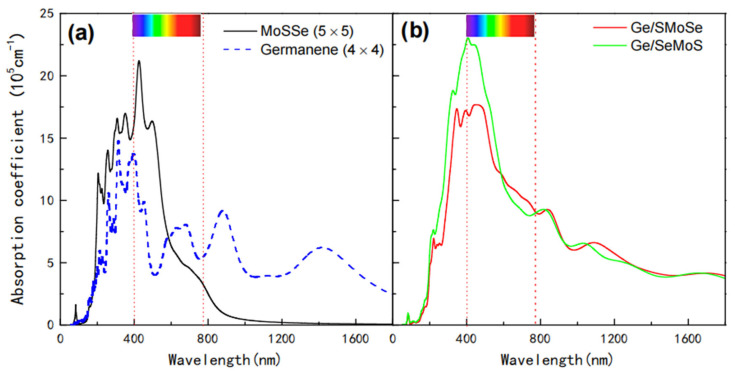
Optical absorption coefficients of (**a**) (4 × 4) germanene and (5 × 5) MoSSe; (**b**) Ge/SMoSe and Ge/SeMoS heterostructures.

To explore potential optical applications of germanene-MoSSe heterostructures, the absorption coefficients for Ge/SMoSe and Ge/SeMoS are also calculated; the results are shown in Figure 4 together with those of free-standing germanene and a pristine MoSSe layer. As clearly shown in Figure 4a, the MoSSe monolayer exhibits strong light absorption ability in UV and visible regions, with the main absorption peak located at ~426 nm. Except in the UV and visible regions, germanene also shows good absorption in the IR region. In Figure 4b, the variations in absorption curves for Ge/SMoSe and Ge/SeMoS heterostructures are similar, which seem to combine the absorption characteristics of the corresponding component layers. Therefore, the heterostructures can exhibit a much wider light absorption ranging from UV to IR. Compared to the MoSSe layer, the enhanced optical absorption of the heterostructure appears in the UV and visible light regions, within the wavelength range from ~350 to ~700 nm, and the highest absorption peak for Ge/SeMoS (at ~406 nm) is higher than that for Ge/SMoSe (at ~446 nm). Moreover, it is also found that the optical absorption properties for both types of heterostructures are not sensitive to the stacking pattern (see Figure S2 from Supplementary Materials).

**Figure 5:**
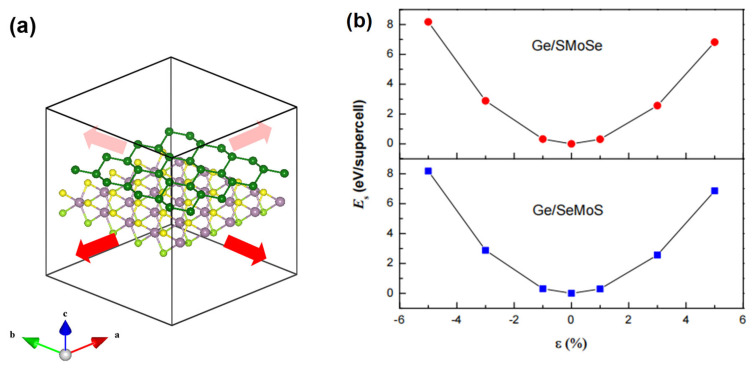
(**a**) The schematic map of Ge/SMoSe heterostructure under in-plane tensile biaxial strain (**b**) The variation of strain energy *E*_s_ as a function of applied biaxial strain ranging from −5% to 5%.

In actual applications, strain deformation is inevitable in heterostructures due to lattice misfit between the different components. On the other hand, the properties of heterostructures can also be well-tuned by applied strains [26,28,29]. Therefore, strain effect on the germanene-MoSSe heterostructures is also considered. An in-plane biaxial strain ε is imposed to the heterostructures through stretching (or compressing) the supercell simultaneously along x and y directions (schematic shown in Figure 5a), which is defined as the following formula:

(3) $$\varepsilon =(a-a_{0})/a_{0}$$

where a and $a_{0}$ are the lattice constants of strained and unstrained heterostructures, and the positive and negative values of ε represent tensile strain and compressive strain, respectively. We firstly explore the total energies of Ge/SMoSe and Ge/SeMoS under the different strains considered. The variation in strain energy $E_{s}$ per supercell as a function of ε is shown in Figure 5b. The almost perfect parabolic curves are obtained under the applied strains for both Ge/SMoSe and Ge/SeMoS heterostructures. That is to say, $E_{s}$ is proportional to the square of ε ranging from −5% to +5%; thus, no essential phase transition in the heterostructures occurs under strain deformation. In the following, we focus on the changes of the Dirac cone, the SBH, as well as optical absorption peaks of the heterostructures under different biaxial strains. 

**Figure 6:**
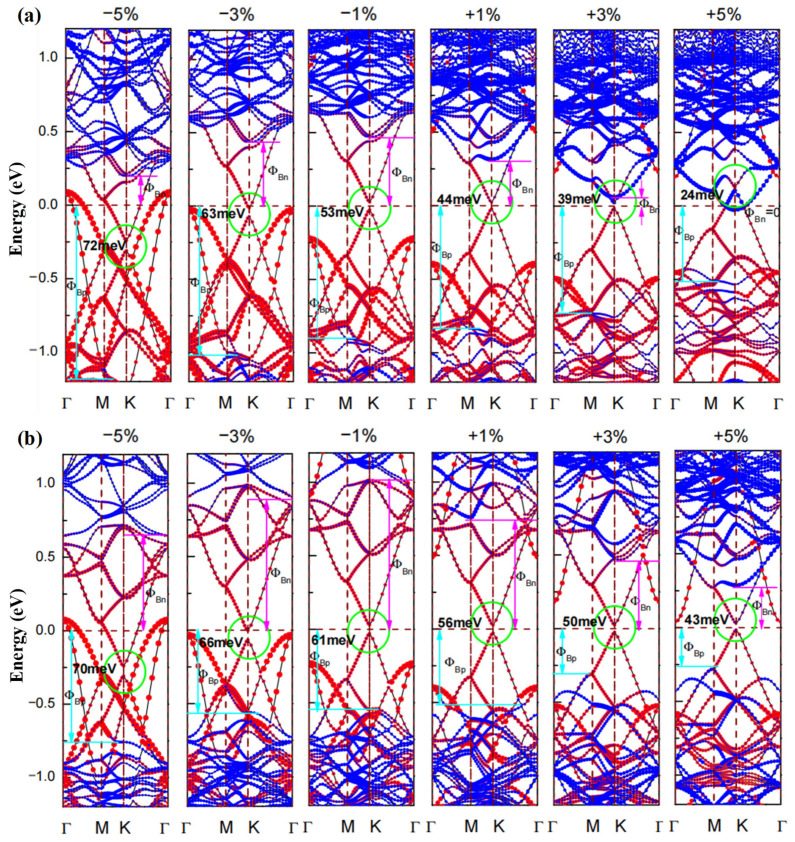
The weighted electronic band structures of (**a**) Ge/SMoSe and (**b**) Ge/SeMoS under different deformation strains, the contributions of MoSSe and germanene are marked by solid blue and red circles, respectively. The Dirac gap is marked with green circle, the n-type and p-type Schottky barrier heights ($\Phi_{\mathrm{Bn}}$ and $\Phi_{\mathrm{Bp}}$ ) are marked with magenta and cyan arrowlines, respectively.

**Figure 7:**
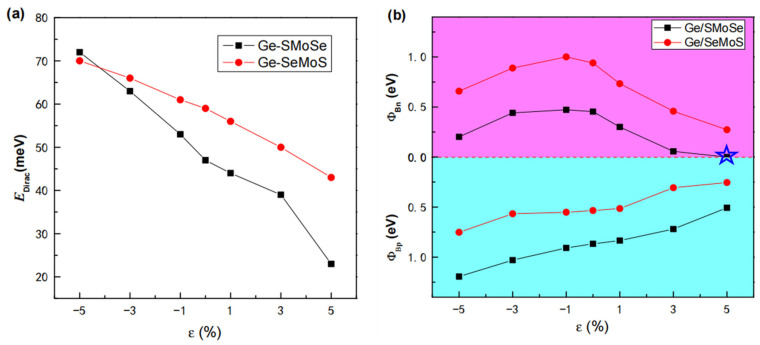
(**a**) The Dirac gaps $E_{\mathrm{Dirac}}$ at *K* point for Ge/SMoSe and Ge/SeMoS heterostructures as a function of strain; (**b**) The SBHs ($\Phi_{\mathrm{Bn}}$ and $\Phi_{\mathrm{Bp}}$ ) of Ge/SMoSe and Ge/SeMoS heterostructures as a function of strain.

Figure 6 gives the weighted electronic band structures of Ge/SMoSe and Ge/SeMoS under different deformation strains. Firstly, we can see the Dirac cone (at K point) of the germanene layer in the heterostructure shifts down to lower energy under compressive strains while it moves toward higher energy under tensile strains; this is similar to what is found in strain-tuned free-standing germanene [54]. In addition, the formed Dirac gap $E_{\mathrm{Dirac}}$ (marked with a green circle in Figure 6) can be well-tuned by the applied strains, with a monotonous decrease as ε varies from −5% to +5%, shown clearly in Figure 7a. Secondly, regardless of whether the heterostructure is under tensile strain or compressive strain, the CBM of the MoSSe layer always moved down with enhancement of the strain deformation, gradually lowering the *n*-type SBH $\Phi_{\mathrm{Bn}}$ (marked in Figure 6 with a magenta arrowline). However, the situation is different for the VBM of the MoSSe layer. It gradually moves up with enhancement of tensile strain, but moves down as the compressive strain increases. That is to say, tensile strain on heterostructures can lower the *p*-type SBH $\Phi_{\mathrm{Bp}}$ (marked in Figure 6 with a cyan arrowline), while compressive strain can uplift $\Phi_{\mathrm{Bp}}$. Therefore, a transition from Schottky to Ohmic behavior is expected when imposing suitable in-plane biaxial strain on heterostructures, and such transition behavior is much easier to achieve by applying tensile strains. In detail, Figure 7b gives the SBHs ($\Phi_{\mathrm{Bn}}$ and $\Phi_{\mathrm{Bp}}$) for Ge/SMoSe and Ge/SeMoS heterostructures under different strains. For instance, we can see that the Schottky–Ohmic transition in Ge/SMoSe happens at a tensile strain of ~5%.

**Figure 8:**
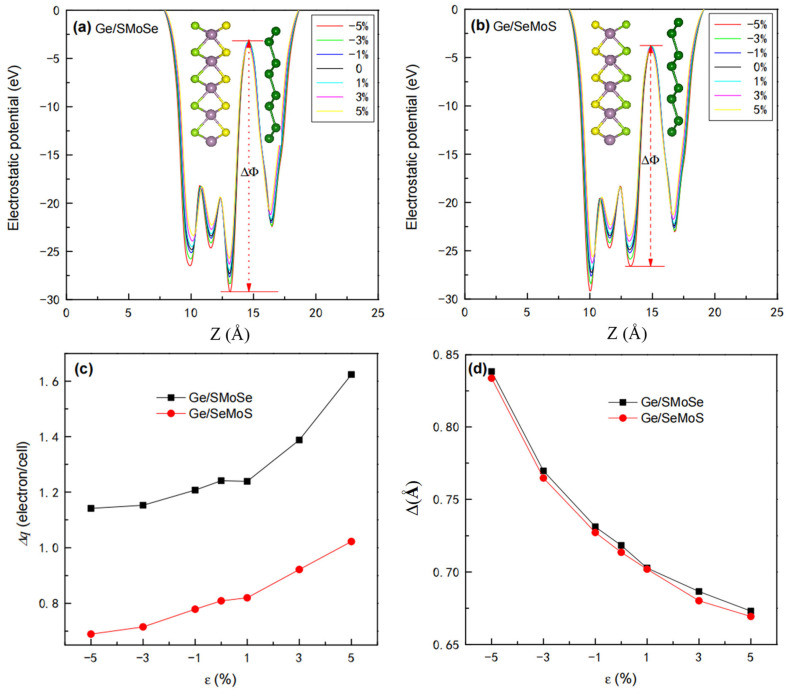
Electrostatic potentials of (**a**,**b**) heterostructures under different strains. Corresponding charge transfers Δq from germanene to the MoSSe layer, buckling of the germanene layer Δ, as functions of strain are given in (**c**,**d**), respectively.

In metal/semiconductor heterojunctions, a low contact resistance is desirable. Thus, a narrow and low interface potential barrier (which can be evaluated as the difference of electrostatic potential between the interlayer gap and the germanene layer) is usually needed. Figure 8a,b illustrate the strain-dependent planar-averaged electrostatic potential for Ge/SMoSe and Ge/SeMoS respectively, in which the interface potential barrier ΔΦ is marked out. From Figure 8a,b, we can see that the heights of the electrostatic potential peaks for two types of heterostructures all gradually reduce with increasing tension, while they increase with enhanced compression; thus, a drop in ΔΦ is observed as ε varies from −5% to +5%. Therefore, it is possible to improve the interface contact of the germanene-MoSSe heterojunction through tensile strains. The change in electrostatic peaks should also lead to charge redistribution in the heterostructures. To better understand this, the strain-dependent charge transfer Δq from germanene to the MoSSe layer is shown in Figure 8c. It can be clearly seen that Δq monotonously increases as ε varies from −5% to +5%. Flattening in the germanene layer suggests enhanced sp^2^ hybridization in the Ge-Ge bond with improved π electron mobility, which could result in a larger charge transfer Δq between the component layers. As expected, the average buckling height of the germanene layer Δ gradually reduces with increasing tension, while it increases with enhancing compression (Figure 8d). This agrees well with the variation trend of Δq under applied strains.

**Figure 9:**
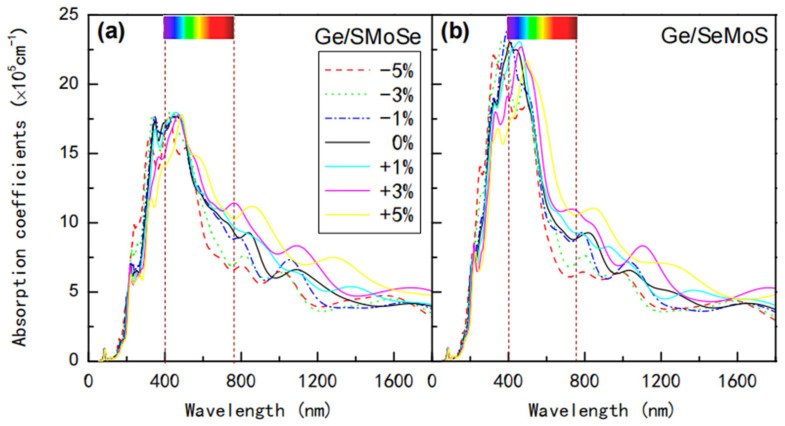
Optical absorption coefficients of (**a**) Ge/SMoSe and (**b**) Ge/SeMoS heterostructures under different strains.

The strain-dependent absorption coefficients of Ge/SMoSe and Ge/SeMoS are also analyzed, and the results are shown in Figure 9a,b. It can be observed that the absorption spectra of heterostructures all gradually shift to longer wavelength (red-shift) as ε varies from −5% to +5%, which leads to an enhanced optical absorption in visible (about 600~800 nm) and IR regions. The strain-tuned optical absorption properties of germanene-MoSSe heterostructures should have potential applications in nanoscale optical sensors. 

## 4. Conclusions

In conclusion, DFT calculations are performed to investigate the electronic and optical absorption properties of 2D vertical heterostructures constructed by Janus MoSSe and germanene. Our results show that a tiny gap can be opened up at the Dirac point in both Ge/SMoSe and Ge/SeMoS heterostructures with intrinsic high-speed carrier mobility of the germanene layer well-preserved. An n-type Schottky contact is formed in Ge/SMoSe, while a p-type one is formed in Ge/SeMoS. The heterostructures can exhibit an extended optical absorption range (including UV, visible and IR light regions) compared to the corresponding individual layers. The position of the Dirac cone, the Dirac gap value as well as the position of the optical absorption peak in both Ge/SMoSe and Ge/SeMoS heterostructures can be tuned by applying in-plane strains. In addition, it is also predicted that a Schottky–Ohmic transition can occur when suitable in-plane strain is imposed (especially by tensile strain) on the heterostructures. These results can provide a helpful guide for designing future nanoscale optoelectronic devices based on germanene-MoSSe vdW heterostructures.

## Figures and Tables

**Table 1:** Calculated binding energies $E_{b}$, lattice constant $a_{0}$, interlayer distance $d_{0}$, buckling height of germanene layer Δ as well as the height of MoSSe layer $h_{s-s_{e}}$.

Type	Configuration	$E_{b}(\mathrm{meV}/{Å}^{2})$	$a_{0}(Å)$	$d_{0}(Å)$	Δ	$h_{s-s_{e}}(Å)$
Ge/SMoSe	Hol	−20.272	16.199	2.924	0.718	3.237
	Top(S)	−20.259	16.202	2.921	0.717	3.237
	Mo	−20.268	16.202	2.920	0.718	3.237
	Bridge	−20.262	16.200	2.926	0.717	3.237
Ge/SeMoS	Hol	−21.746	16.195	3.067	0.714	3.238
	Top(Se)	−21.730	16.196	3.071	0.713	3.238
	Mo	−21.769	16.196	3.068	0.712	3.238
	Bridge	−21.720	16.197	3.064	0.714	3.238

## Data Availability

The data presented in this study are available on request from the corresponding author.

## Supplementary Materials

The following are available online at https://www.mdpi.com/article/10.3390/nano12193498/s1, Figure S1: The weighted band structures for different configurations of (a) Ge/SMoSe and (b) Ge/SeMoS het-erostructures. The contributions from MoSSe layer and germanene are marked by solid blue and red circles respectively; Figure S2: Optical absorption coefficients for different configurations of (a) Ge/SMoSe and (b) Ge/SeMoS heterostructures.

## References

[1] Novoselov K.S., Geim A.K., Morozov S.V., Jiang D., Zhang Y., Dubonos S.V., Grigorieva I.V., Firsov A.A. (2004). Electric field effect in atomically thin carbon films. Science.

[2] Miró P., Audiffred M., Heine T. (2014). An atlas of two-dimensional materials. Chem. Soc. Rev..

[3] Riis-jensen A.C., Deilmann T., Olsen T., Thygesen K.S. (2019). Classifying the Electronic and Optical Properties of Janus Monolayers. ACS Nano.

[4] Zhang L., Yang Z., Gong T., Pan R., Wang H., Guo Z., Zhang H., Fu X. (2020). Recent advances in emerging Janus two-dimensional materials: From fundamental physics to device applications. J. Mater. Chem. A.

[5] Hou B., Zhang Y., Zhang H., Shao H., Ma C., Zhang X., Chen Y., Ni G., Zhu H. (2020). Room Temperature Bound Excitons and Strain-Tunable Carrier Mobilities in Janus Monolayer Transition-Metal Dichalcogenides. J. Phys. Chem. Lett..

[6] Yin W.-J., Tan H.-J., Ding P.-J., Wen B., Li X.-B., Teobaldi G., Liu L.-M. (2021). Recent advances in low-dimensional Janus materials: Theoretical and simulation perspectives. Mater. Adv..

[7] Cheng Y.C., Zhu Z.Y., Tahir M., Schwingenschlögl U. (2013). Spin Orbit-Induced Spin Splittings in Polar Transition Metal Dichalcogenide Monolayers. EPL.

[8] Dong L., Lou J., Shenoy V.B. (2017). Large In-Plane and Vertical Piezoelectricity in Janus Transition Metal Dichalchogenides. ACS Nano.

[9] Yagmurcukardes M., Sevik C., Peeters F.M. (2019). Electronic, vibrational, elastic, and piezoelectric properties of monolayer Janus MoSTe phases: A first-principles study. Phys. Rev. B Condens. Matter.

[10] Ji Y., Yang M., Lin H., Hou T., Wang L., Li Y., Lee S.-T. (2018). Janus Structures of Transition Metal Dichalcogenides as the Heterojunction Photocatalysts for Water Splitting. J. Phys. Chem. C.

[11] Ju L., Bie M., Shang J., Tang X., Kou L. (2020). Janus transition metal dichalcogenides: A superior platform for photocatalytic water splitting. J. Phys. Mater..

[12] Lu A.-Y., Zhu H., Xiao J., Chuu C.-P., Han Y., Chiu M.-H., Cheng C.-C., Yang C.-W., Wei K.-H., Yang Y. (2017). Janus monolayers of transition metal dichalcogenides. Nat. Nanotechnol..

[13] Zhang J., Jia S., Kholmanov I., Dong L., Er D., Chen W., Guo H., Jin Z., Shenoy V.B., Shi L. (2017). Janus monolayer transition-metal dichalcogenides. ACS Nano.

[14] Yin W.-J., Wen B., Nie G.-Z., Wei X.-L., Liu L.-M. (2018). Tunable dipole and carrier mobility for a few layer Janus MoSSe structure. J. Mater. Chem. C.

[15] Ma X., Yong X., Jian C.-C., Zhang J. (2019). Transition Metal-Functionalized Janus MoSSe Monolayer: A Magnetic and Efficient Single-Atom Photocatalyst for Water-Splitting Applications. J. Phys. Chem. C.

[16] Jin C., Tang X., Tan X., Smith S.C., Dai Y., Kou L. (2019). A Janus MoSSe monolayer: A superior and strain-sensitive gas sensing material. J. Mater. Chem. A.

[17] Chaurasiya R., Dixit A. (2019). Defect engineered MoSSe Janus monolayer as a promising two dimensional material for NO_2_ and NO gas sensing. Appl. Surf. Sci..

[18] Wu P., Cui Z., Li Q., Ding Y. (2021). Gas (CO and NO) adsorption and sensing based on transition metals functionalized Janus MoSSe. Appl. Surf. Sci..

[19] Shang C., Lei X., Hou B., Wu M., Xu B., Liu G., Ouyang C. (2018). Theoretical Prediction of Janus MoSSe as a Potential Anode Material for Lithium-Ion Batteries. J. Phys. Chem. C.

[20] Tang X., Ye H., Liu W., Liu Y., Guo Z., Wang M. (2021). Lattice-distorted lithiation behavior of a square phase Janus MoSSe monolayer for electrode applications. Nanoscale Adv..

[21] Xia W., Dai L., Yu P., Tong X., Song W., Zhang G., Wang Z. (2017). Recent progress in van der Waals heterojunctions. Nanoscale.

[22] Liao W., Huang Y., Wang H., Zhang H. (2019). Van der Waals heterostructures for optoelectronics: Progress and prospects. Appl. Mater. Today.

[23] Cai Z., Liu B., Zou X., Cheng H.-M. (2018). Chemical Vapor Deposition Growth and Applications of Two-Dimensional Materials and Their Heterostructures. Chem. Rev..

[24] Ju L., Bie M., Zhang X., Chen X., Kou L. (2021). Two-dimensional Janus van der Waals heterojunctions: A review of recent research progresses. Front. Phys..

[25] Idrees M., Din H.U., Ali R., Rehman G., Hussain T., Nguyen C.V., Ahmad I., Amin B. (2019). Optoelectronic and solar cell applications of janus monolayers and their van der waals heterostructures. Phys. Chem. Chem. Phys..

[26] Chen D., Lei X., Wang Y., Zhong S., Liu G., Xu B., Ouyang C. (2019). Tunable electronic structures in BP/MoSSe van der Waals heterostructures by external electric field and strain. Appl. Surf. Sci..

[27] Yin W., Wen B., Ge Q., Zou D., Xu Y., Liu M., Wei X., Chen M., Fan X. (2019). Role of intrinsic dipole on photocatalytic water splitting for Janus MoSSe/nitrides heterostructure: A first-principles study. Prog. Nat. Sci..

[28] Deng S., Li L., Rees P. (2019). Graphene/MoXY Heterostructures Adjusted by Interlayer Distance, External Electric Field, and Strain for Tunable Devices. ACS Appl. Nano Mater..

[29] Yu C., Cheng X., Wang C., Wang Z. (2019). Tuning the n-type contact of graphene on janus MoSSe monolayer by strain and electric field. Phys. E.

[30] Wang Y., Chen R., Luo X., Liang Q., Wang Y., Xie Q. (2022). First-Principles Calculations on Janus MoSSe/Graphene van der Waals Heterostructures: Implications for Electronic Devices. ACS Appl. Nano Mater..

[31] Zhou S.-H., Zhang J., Ren Z.-Z., Gu J.-F., Ren Y.-R., Huang S., Lin W., Li Y., Zhang Y.-F., Chen W.-K. (2020). First-principles study of MoSSe_graphene heterostructures as anode for Li-ion batteries. Chem. Phys..

[32] Lin H., Lou N., Yang D., Jin R., Huang Y. (2021). Janus MoSSe/graphene heterostructures: Potential anodes for lithium-ion batteries. J. Alloys Compd..

[33] Cahangirov S., Topsakal M., Aktürk E., Şahin H., Ciraci S. (2009). Two- and one-dimensional honeycomb structures of silicon and germanium. Phys. Rev. Lett..

[34] Liu N., Bo G., Liu Y., Xu X., Du Y., Dou S.X. (2019). Recent Progress on Germanene and Functionalized Germanene: Preparation, Characterizations, Applications, and Challenges. Small.

[35] Ye X., Shao Z., Zhao H., Yang L., Wang C. (2014). Intrinsic carrier mobility of germanene is larger than graphene’s: First-principle calculations. RSC Adv..

[36] Liu C.-C., Jiang H., Yao Y.G. (2011). Low-energy effective Hamiltonian involving spin-orbit coupling in silicene and two-dimensional germanium and tin. Phys. Rev. B Condens. Matter.

[37] Bandaru P.R., Pichanusakorn P. (2010). An outline of the synthesis and properties of silicon nanowires. Semicond. Sci. Technol..

[38] Hussain T., Kaewmaraya T., Chakraborty S., Vovusha H., Amornkitbamrung V., Ahuja R. (2018). Defected and functionalized germanene-based nanosensors under sulfur comprising gas exposure. ACS Sens..

[39] Pang Q., Zhang C.-L., Li L., Fu Z.-Q., Wei X.-M., Song Y.-L. (2014). Adsorption of alkali metal atoms on germanene: A first-principles study. Appl. Surf. Sci..

[40] Pang Q., Li L., Zhang C.-L., Wei X.-M., Song Y.-L. (2015). Structural, electronic and magnetic properties of 3d transition metal atom adsorbed germanene: A first-principles study. Mater. Chem. Phys..

[41] Zhou S., Zhao J. (2016). Electronic structures of germanene on MoS_2_: Effect of substrate and molecular adsorption. J. Phys. Chem. C.

[42] Pang Q., Xin H., Gao D.-L., Zhao J., Chai R.-P., Song Y.-L. (2021). Strain effect on the electronic and optical properties of Germanene/MoS_2_ heterobilayer. Mater. Today Commun..

[43] Cai Y., Chuu C.-P., Wei C.M., Chou M.Y. (2013). Stability and electronic properties of two-dimensional silicene and germanene on graphene. Phys. Rev. B Condens. Matter..

[44] Persichetti L., Jardali F., Vach H., Sgarlata A., Berbezier I., De Crescenzi M., Balzarotti A. (2016). Van der Waals Heteroepitaxy of Germanene Islands on Graphite. J. Phys. Chem. Lett..

[45] Kresse G., Furthmüller J. (1996). Efficiency of *ab–initio* total energy calculations for metals and semiconductors using a plane–wave basis set. Comput. Mater. Sci..

[46] Kresse G., Joubert D. (1999). From ultrasoft pseudopotentials to the projector augmented-wave method. Phys. Rev..

[47] Perdew J.P., Burke K., Ernzerhof M. (1996). Generalized gradient approximation made simple. Phys. Rev. Lett..

[48] Grimme S. (2006). Semiempirical GGA-type density functional constructed with a long-range dispersion correction. J. Comput. Chem..

[49] Monkhorst H.J., Pack J.D. (1976). Special points for Brillouin-zone integrations. Phys. Rev. B.

[50] Gajdoš M., Hummer K., Kresse G., Furthmüller J., Bechstedt F. (2006). Linear optical properties in the projector-augmented wave methodology. Phys. Rev. B Condens. Matter.

[51] Björkman T., Gulans A., Krasheninnikov A.V., Nieminen R.M. (2012). van der Waals Bonding in Layered Compounds from Advanced Density-Functional First-Principles Calculations. Phys. Rev. Lett..

[52] Bardeen J. (1974). Surface states and rectification at a metal semi-conductor contact. Phys. Rev..

[53] Bader R. (1990). Atoms in Molecules: A Quantum Theory.

[54] Wang Y., Ding Y. (2013). Strain-induced self-doping in silicene and germanene from first-principles. Solid State Commun..

