# Supplementary material for: In-Plane Strain Tuned Electronic and Optical Properties in Germanene-MoSSe Heterostructures
Source: Nanomaterials (Basel). 2022 Oct 6;12(19):3498. doi: 10.3390/nano12193498 (PMC9565274; doi:10.3390/nano12193498)
Supplement: Supplementary file 1 [file nanomaterials-12-03498-s001.zip › nanomaterials-1927072-supplementary.pdf]

nanomaterials

Supplementary Materials

In-Plane Strain Tuned Electronic and Optical Properties in Germanene-MoSSe Heterostructures

Qing Pang [1,*], Hong Xin [1], Ruipeng Chai [1], Dangli Gao [1,2,*], Jin Zhao [1], You Xie [3] and Yuling Song [4]

[1] College of Science, Xi'an University of Architecture and Technology, Xi'an 710055, China
[2] Shaanxi Key Laboratory of Nano Materials and Technology, Xi'an University of Architecture and Technology, Xi'an 710055, China
[3] College of Science, Xi'an University of Science and Technology, Xi'an 710054, China
[4] College of Physics and Electronic Engineering, Nanyang Normal University, Nanyang 473061, China
* Correspondence: pangqinglxy@xauat.edu.cn (Q.P.); gaodangli@xauat.edu.cn (D.G.)

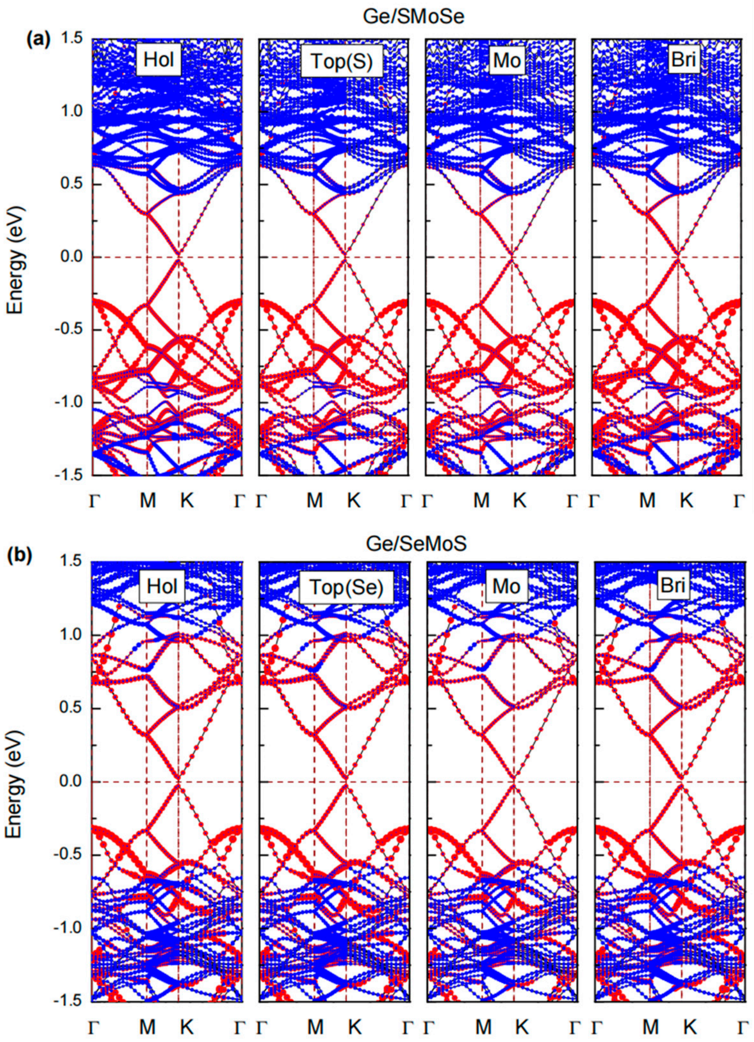

Figure S1. The weighted band structures for different configurations of (**a**) Ge/SMoSe and (**b**) Ge/SeMoS heterostructures. The contributions from MoSSe layer and germanene are marked by solid blue and red circles respectively.

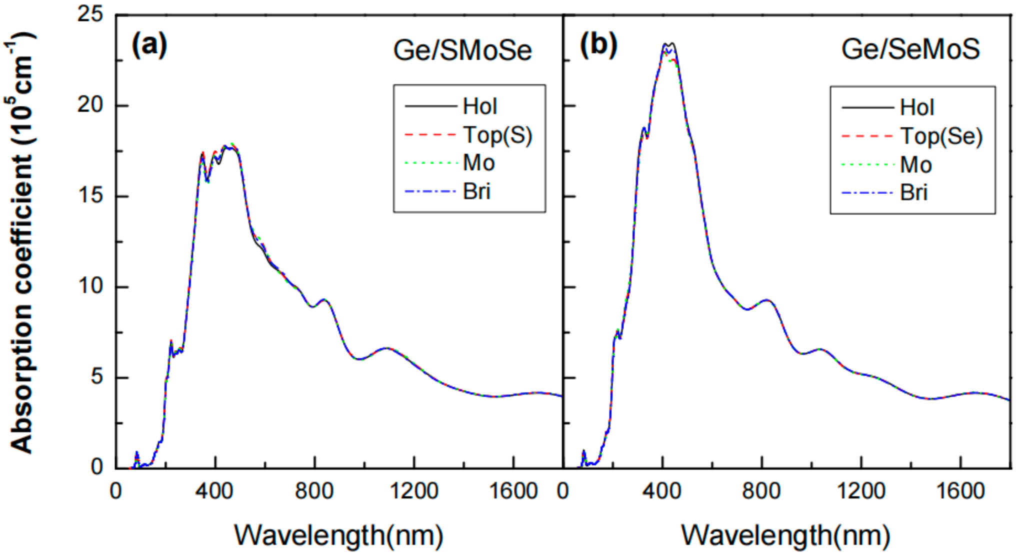

Figure S2. Optical absorption coefficients for different configurations of (**a**) Ge/SMoSe and (**b**) Ge/SeMoS heterostructures.
